# Competitive growth experiments with a high-lipid *Chlamydomonas reinhardtii* mutant strain and its wild-type to predict industrial and ecological risks

**DOI:** 10.1186/s13568-016-0305-x

**Published:** 2017-01-03

**Authors:** David A. Russo, Andrew P. Beckerman, Jagroop Pandhal

**Affiliations:** 1 AlgaeCytes Ltd., Discovery Park House, Discovery Park, Ramsgate Road, Sandwich, CT13 9ND UK; 2 Department of Animal and Plant Sciences, University of Sheffield, Alfred Denny Building, Western Bank, Sheffield, S10 2TN UK; 3 Department of Chemical and Biological Engineering, University of Sheffield, Mappin Street, Sheffield, S1 3JD UK

**Keywords:** Competition, *Chlamydomonas reinhardtii*, Environmental risk, Lipids, Microalgae, Response surface methodology

## Abstract

Key microalgal species are currently being exploited as biomanufacturing platforms using mass cultivation systems. The opportunities to enhance productivity levels or produce non-native compounds are increasing as genetic manipulation and metabolic engineering tools are rapidly advancing. Regardless of the end product, there are both environmental and industrial risks associated to open pond cultivation of mutant microalgal strains. A mutant escape could be detrimental to local biodiversity and increase the risk of algal blooms. Similarly, if the cultivation pond is invaded by a wild-type (WT) microalgae or the mutant reverts to WT phenotypes, productivity could be impacted. To investigate these potential risks, a response surface methodology was applied to determine the competitive outcome of two *Chlamydomonas reinhardtii* strains, a WT (CC-124) and a high-lipid accumulating mutant (CC-4333), grown in mixotrophic conditions, with differing levels of nitrogen and initial WT to mutant ratios. Results of the growth experiments show that mutant cells have double the exponential growth rate of the WT in monoculture. However, due to a slower transition from lag phase to exponential phase, mutant cells are outcompeted by the WT in every co-culture treatment. This suggests that, under the conditions tested, outdoor cultivation of the *C. reinhardtii* cell wall-deficient mutant strains does not carry a significant environmental risk to its WT in an escape scenario. Furthermore, lipid results show the mutant strain accumulates over 200% more TAGs per cell, at 50 mg L^−1^ NH_4_Cl, compared to the WT, therefore, the fragility of the mutant strain could impact on overall industrial productivity.

**Electronic supplementary material:**

The online version of this article (doi:10.1186/s13568-016-0305-x) contains supplementary material, which is available to authorized users.

## Introduction

Research into microalgal biotechnology has progressed considerably in the last few decades, with successful commercial examples of large-scale production in outdoor raceway ponds (Borowitzka and Moheimani 2013). Attempts are being made to increase productivity through process optimisation and, increasingly, through strain manipulation, which also enables a diversification in the portfolio of compounds that can be produced. One way to improve productivity of microalgal strains for biotechnology is through random mutagenesis, followed by screening for desired characteristics (Guarnieri and Pienkos 2015; Work et al. 2010). Although mutagenesis and screening can be time consuming and costly, it can improve metabolite production several fold. One species where this has been successfully demonstrated is *Chlamydomonas reinhardtii* (Li et al. 2010).

*C. reinhardtii* is a popular model organism with a fully sequenced and well-annotated genome (Merchant et al. 2007). Moreover, there are also powerful molecular tools available to modify and characterise its genome (Jinkerson and Jonikas 2015). It is, therefore, commonly used in laboratory scale microalgal biotechnology research and development. Like other microalgal species, *C. reinhardtii* responds to stress such as nitrogen deprivation (Siaut et al. 2011) with an accumulation of reserve molecules (e.g. lipid, starch). Recently, studies have found that inhibiting starch synthesis in *C. reinhardtii*, via mutagenesis, can divert the carbon flow towards an increase in intracellular triglycerides (TAGs) (Li et al. 2010; Work et al. 2010). These mutants can accumulate up to 3.5 times more TAGs than the wild-type (WT) strain under nutrient stress conditions (Li et al. 2010). These are promising advances, particularly in the field of microalgal biodiesel research, where process economics remain unfavourable and hence productivity increases through technological advances are keenly sought. However, to scale up production of TAGs through laboratory generated microalgal strains, such as the *C. reinhardtii* low-starch mutant, there are many challenges that need to be overcome, including appropriate strain cultivation strategies, efficient implementation of nutrient deprivation stress as well as low cost harvesting.

Here two risks associated with large-scale cultivation of microalgal mutant strains are assessed by undertaking a series of competitive growth and productivity experiments. The first is defined as an environmental risk, with the release of genetically altered organisms, which could impact on the biodiversity of the surrounding environment by outcompeting native strains (Snow and Smith 2012). This could further influence the community structure of aquatic ecosystems if the strain generates direct or indirect effects on microbes, grazers or higher trophic levels (invertebrate and vertebrate), ultimately influencing the probability of algae blooms or altered microbial community function (Flynn et al. 2013). Currently, there are no recommended laboratory protocols that can be used to infer or guide the level of risk associated with the escape of specific mutant algal strains into the environment. However, there are environmental risk assessments being generated which can be used for guidance, for example, decision trees asking questions such as: Does the mutant strain survive outside the bioreactor/raceway pond? Does it compete or interact with the WT? (Jeremy Sweet, personal communication).

In addition to the environmental risk, there is also an industrial risk, where the benefit of reduced starch synthesis (Li et al. 2010; Zabawinski et al. 2001) is lost during the cultivation process, and hence the enhanced TAG accumulation during nutrient stress is not realised. This could occur through natural mutation in the genes altered by random mutagenesis, or in the case of a deletion mutation, mating with an invasive WT strain. This could lead to a strain with TAG productivity characteristics similar to the WT dominating the outdoor raceway pond, hence, reducing overall TAG productivity. Moreover, raceway contamination through invasion by a local microalgal species, with lower TAG accumulation levels, is also an industrial risk.

Such risks would benefit from a strategic investigation of competitive dynamics where the fitness of modified or mutant strains are determined against their WT as a baseline. Although conducted in the laboratory, at small scale and in less complex conditions relative to the environment, it can provide key clues to estimate potential risks. If the fitness of the transgenic strain is relatively lower than that of the WT, then it can be argued that any environmental risk analysis could be based solely on the risks of cultivating the WT (Gressel et al. 2013). In this scenario, the industrial risk of reduced productivity, due to WT overtaking the transgenic strain, would need to be estimated.

To further this objective, the competitive outcome between cultivating two *C. reinhardtii* strains, the CC-124 strain (hereon referred to as WT strain) and the starchless CC-4333 strain (Zabawinski et al. 2001), (hereon referred to as mutant strain) was explored. An initial monoculture assessment of growth was undertaken followed by competing the strains against each other. A response surface competition experiment was developed, varying both the initial proportion of the WT and mutant strains, together with the nitrogen (ammonium chloride, NH_4_Cl) concentration in the media. An assessment of both environmental risk during an “escape scenario” and the industrial risk associated with being potentially out competed by the WT strain was made using this design. Subsequently, the industrial risk was further analysed by assessing total biomass accumulation and TAG productivity, again in response to varying the initial proportion of the strains and NH_4_Cl concentration.

## Methods

### Strains and culturing conditions

CC-124 is a WT *C. reinhardtii* strain with a cell wall and flagella, widely used in laboratory studies (Pröschold et al. 2005) and CC-4333 is a low starch, no flagellum, cell wall-deficient mutant, obtained through insertional mutagenesis, which lacks the catalytic (small) subunit of ADP-glucose pyrophosphorylase (Ball et al. 1991). Both strains were obtained from the Chlamydomonas Resource Center (University of Minnesota, USA). The cultures were maintained in mixotrophic conditions on tris-acetate-phosphate (TAP) medium as described by Gorman and Levine (1965). Cells were grown in 50 mL centrifuge tubes, with 25 mL of culture, and under 70 μmol m^−2^ s^−1^ constant illumination on an orbital shaker at 110 rpm. Constant illumination and mixotrophic growth conditions could, potentially, limit any comparison with natural environments and industrial cultivation under natural light. However, the vast majority of lipid studies having been undertaken in these conditions (e.g. Work et al. 2010; Ramanan et al. 2013), therefore, they were chosen in order to serve as a point of comparison.

### Competition experiment

The response surface design is an experimental design where two variables are varied simultaneously, and estimates made of response variables at several combinations. A standard analysis for such data is to fit a response surface model (RSM), the basic fitting a second order polynomial for each variable, and an interaction term between them. This is a flexible model that can estimate planes, ridges, valleys and peaks as a function of linear polynomial functions of each variable, and their interaction. It specifically allows us to evaluate whether there are additive or interactive (synergistic/antagonistic) effects of competition and nutrient enrichment on, for example, TAG production or long term dynamics (e.g. competitive outcomes).

In our RSM design, cultures were grown to late log phase, harvested by centrifugation and resuspended at 1 × 10^5^ cells mL^−1^, in parallel, in TAP medium with four different concentrations of NH_4_Cl: 50, 100, 200 and 375 mg L^−1^ and five different initial WT:mutant cell number ratios (100:0, 75:25, 50:50, 75:25 and 0:100). 200 μL aliquots were taken immediately after inoculation (0 h), and every 24 h after that, for cell counts and TAG quantification. A total of 1.2 mL was removed, from each tube, over the course of the experiment. Cell counts were performed using a Bright-Line glass haemocytometer (Hausser Scientific, USA)) on a BX 51 microscope (Olympus, Japan). The carrying capacities (*K*) and exponential growth rates (μ) were estimated by fitting a logistic growth curve to each respective cell count time series in GraphPad Prism 6.07 (GraphPad Software, Inc., USA). The WT and mutant strains are visually undistinguishable. Therefore, to separately count each strain the total number of cells were counted, samples were then incubated, for 5 min in 0.5% Triton X-100 (Sigma, USA), to completely lyse the cell wall deficient mutant cells. Preliminary tests with all initial WT:mutant cell number ratios showed a correlation of 0.98 between theoretical and observed cell lysis values (Additional file 1: Fig. A1). Afterwards, the total number of cells were recounted and the difference between both counts equalled the number of mutant cells present. Cells for TAG quantification were lysed by sonication on ice with a FB 15051 sonicator (Fischer Scientific, USA), at power level three and duty cycle 30%, for three cycles of 15 s. TAGs were then quantified with the commercially available Thermo Scientific Infinity TAG reagent kit (Thermo Scientific, USA) according to manufacturer’s recommendations.

From the time series of the three replicates of each NH_4_Cl and initial WT:mutant cell number ratio treatment combination *K* was estimated and used to fit and visualise the RSM. The RSM models and visualisations were fit in the R Statistical Programming Environment (R Development Core Team 2014) by employing the package “rsm” (Lenth 2009).

## Results

### WT and mutant *C. reinhardtii* strains grown in monoculture

**Fig. 1:**
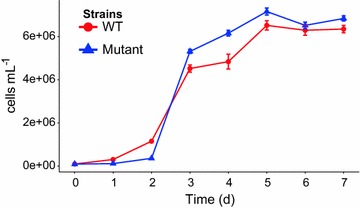
Growth curves, measured in cells mL^−1^, of WT (CC-124) and mutant (CC-4333) *C.* *reinhardtii* strains grown in nutrient replete conditions. *Triangles* indicate the mutant strain and *circles* indicate the WT strain

Our initial focus was to characterise and compare the growth patterns of both strains in monoculture. When grown in monoculture in 375 mg L^−1^ NH_4_Cl, the WT strain has an μ of 1.76 ± 0.26 day^−1^, calculated between days 2 and 3, and achieves a *K* of 6.25 × 10^6^ cells after 5 days (Fig. 1). The mutant strain has a higher, albeit not significantly, maximum cell number, *K*, of 6.68 × 10^6^ cells under these conditions. However, the mutant cells, between days 2 and 3, had a significantly higher μ of 4.08 ± 0.52 day^−1^ (p = 0.008) (Fig. 1), indicating more than double the exponential growth rate of the WT strain. However, it was also observed that although the WT and mutant strains share similar growth patterns, there is a slower transition from lag phase to exponential phase with the mutant strain. This was confirmed by a significantly lower growth rate of mutant cells in the first 2 days of the experiment, 0.68 ± 0.04 day^−1^ (p = 0.008), when compared to the growth rate of the WT in the same timeframe, 1.24 ± 0.01 day^−1^.

### WT and mutant strain growth competition as a function of NH_4_Cl concentrations and initial WT:mutant cell number ratio

**Fig. 2:**
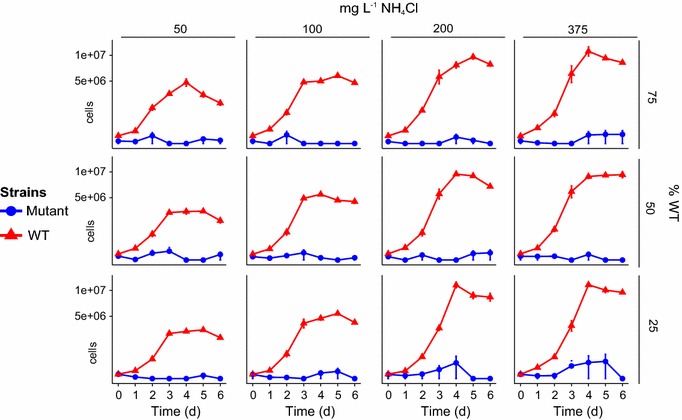
Time series of WT and mutant strain cell numbers as a function of NH_4_Cl concentrations (*horizontal axis*) and percentage of WT cells in initial culture (*vertical axis*). *Triangles* indicate the mutant strain and *circles* indicate the WT strain. Data have been transformed with a square root transformation

In every experimental treatment where the WT and mutant were mixed, the WT strain showed a significantly higher μ and *K* than the mutant strain (Fig. 2). This suggests that the overall fitness of the mutant strain is inferior to that of its WT, when cultivated in co-culture in the conditions tested (i.e. initial ratios, nitrogen concentrations, cultivation conditions etc.). In situations where both strains are sharing the same resource, the WT strain appears to maintain a higher fitness, which prevents the mutant strain from achieving cell numbers that can put the existence of the WT at risk.

### Biomass production as a function of NH_4_Cl concentrations and initial WT:mutant cell number ratio

A RSM was fit to data on the maximum biomass (*K*, estimated from each of the three replicate time series). *K* was estimated by fitting a logistic curve to each time series resulting in three estimates of *K* for each combination of NH_4_Cl concentration and initial WT:mutant cell number ratios. In order to investigate the relationship between the factors, a regression analysis was applied. The results are listed in Table 1 with the multiple correlation coefficient *R* ^*2*^ of 0.861 suggesting that the quadratic polynomial model was suitable for revealing these relationships. As shown in Table 1, *K* varied as a quadratic function of NH_4_Cl concentrations and initial WT:mutant cell number ratio (p < 0.001) and the effect of nutrient levels depended on initial WT:mutant cell number ratios (interaction; p < 0.001).

**Table 1:** Estimated regression coefficients for the RSM fit to data on the variation of *K*

Factor	Estimate	Std. Error	*t*-value	Pr(>|t|)
(Intercept)	−9.5967 × 10^5^	9.6840 × 10^5^	−0.9899	0.3267
NH_4_Cl	1.0607 × 10^5^	9.4882 × 10^3^	11.1789	<0.001
WT:mutant	−8.9544 × 10^6^	2.4137 × 10^6^	−3.7099	<0.001
NH_4_Cl * WT:mutant	−3.0166 × 10^4^	5.0928 × 10^3^	−5.9233	<0.001
NH_4_Cl^2^	−1.5301 × 10^2^	2.0594 × 10^1^	−7.4300	<0.001
WT:mutant^2^	1.0648 × 10^7^	2.1386 × 10^6^	4.9788	<0.001

R^2^: 0.861; R_adj_^2^: 0.8481. _F5,54_ = 66.88, p < 0.001

**Fig. 3:**
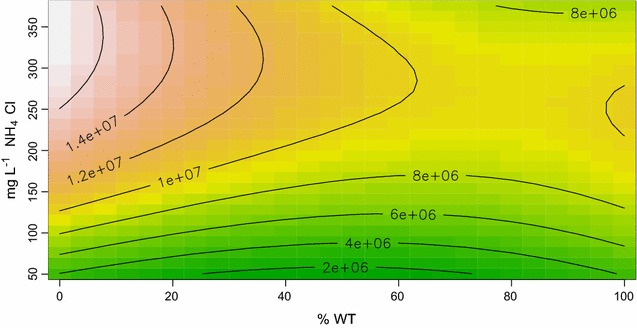
RSM analysis of carrying capacity (*K*) as a function of NH_4_Cl concentrations (*vertical axis*) and percentage of WT cells in initial culture (*horizontal axis*)

The data support a ridge like pattern of *K* (Fig. 3). Expectedly, *K* is highest with the mutant monoculture at higher nutrient levels, although there was no significant difference between the *K* at 200 mg L^−1^ NH_4_Cl and 375 mg L^−1^ NH_4_Cl. The ridge of maximum *K* then decreases with increasing percentage of initial WT.

### TAG production as a function of NH_4_Cl concentrations and initial WT:mutant cell number ratio

**Fig. 4:**
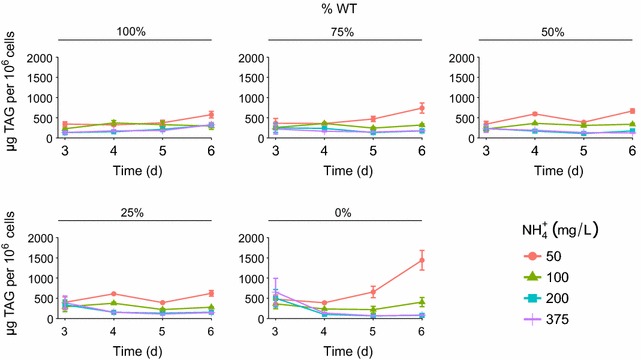
Time series of TAG concentrations per million cells (μg TAG per 10^6^ cells) for each combination of NH_4_Cl concentration and percentage of WT cells in initial culture. *Circles*, *triangles*, *squares* and *vertical lines* indicate 50, 100, 200 and 375 mg L^−1^ NH_4_^+^, respectively

The highest TAG accumulation per million cells, during the stationary phase, occurs with the 100% mutant strain at the lowest nitrogen concentration (50 mg L^−1^ NH_4_Cl). In the mutant and WT co-cultures the results show that, from the perspective of TAGs per million cells, and due to the success of WT growth within the co-cultures, the lipid productivity could be reduced up to 60% (Fig. 4).

## Discussion

The fact that the mutant cells, in exponential phase, had a significantly higher μ of 4.08 ± 0.52 day^−1^ (p = 0.008) (Fig. 1) would suggest that the mutant strain could outcompete the WT strain during an escape scenario presenting an environmental risk. It also hints that there would be a reduced industrial risk from the mutant strain being outcompeted by cells that have reverted to WT lipid accumulation characteristics. In a previous study that compared growth and lipid accumulation in a *C. reinhardtii* WT and the same mutant strain as this study (Work et al. 2010), the highest cell concentration and growth rate were achieved in the mutant strain, and this was attributed to its smaller cell diameter compared to the WT. This observation was confirmed in our study (Additional file 1: Fig. A2) and previously where it is has been demonstrated that cell size and growth rates are negatively correlated (Schlesinger et al. 1981). It was also observed that there is a slower transition from lag phase to exponential phase with the mutant strain compared to the WT. This difference could be detrimental to the survival of the mutant strain when grown in co-culture with the WT strain. It should be noted that this study was performed in a closed system with uniform resource conditions, without the presence of predators (e.g. zooplankton), symbiotic and antagonistic organisms and without fluctuating environmental conditions. Any of these factors that would favour the mutant or WT strain could create an unbalance and allow one to dominate the other.

In order to decipher which of the predicted scenarios from interpreting the monoculture results above were most likely to take place, co-culture experiments were undertaken where equal initial amounts of WT and mutant strain were competed (50:50), followed by a dominating mutant (25:75) and dominating WT (75:25) experiment. Pure cultures were cultivated as controls and the concentration of nitrogen was also varied (NH_4_Cl: 50, 100, 200 and 375 mg L^−1^). Recent reports have hypothesised that genetic mutations of biotechnological interest (e.g. leading to increased lipid content) are all in the opposite direction of evolution (i.e. optimal growth under natural conditions) and therefore should be outcompeted in growth by the respective WT strains (Flynn et al. 2010; Gressel et al. 2013). The results in this study support this theory. Our results showed that when the WT and mutant were mixed, the overall fitness of the mutant strain is inferior to that of its WT (Fig. 2). Work et al. (2010) suggest several reasons why this would happen: under nitrogen deprivation, starchless mutants metabolise less acetate and have severely attenuated levels of photosynthetic O_2_ evolution compared to the WT. This indicates that the mutant strain responds to nitrogen deprivation by decreasing its overall anabolic processes (Work et al. 2010). If the cells in the lag phase of a mixed culture are under similar stress, it could explain why the WT dominates over the mutant in every experimental scenario tested.

Although the competition results showed a clear difference between the WT and mutant, this study was done in a closed system with uniform resource conditions, without the presence of predators (e.g. zooplankton), symbiotic and antagonistic organisms and without fluctuating environmental conditions. Any of these factors that would favour the mutant strain could create an unbalance and allow it to dominate over its WT. For example, in a natural system, differences in grazer palatability can have a dramatic effect on the competitive outcome. Van Donk (1997) performed grazing experiments to determine the role of cell wall structure and nutrient limitation on the digestibility of the *C. reinhardtii* WT and cell wall-deficient mutant. The study revealed that under nutrient limiting conditions, *Daphnia magna* clearance rates of *C. reinhardtii* WT cells were severely debilitated. In contrast, under both non-limiting and limiting nutrient conditions, *C. reinhardtii* mutant cells were cleared equally (Van Donk 1997). This further supports our prediction that mutant cells remain unlikely to outcompete their WT strain and much less so under nutrient stress conditions necessary to take advantage of the mutation.

Industrially, both the data from this study and Van Donk (1997) suggests that outdoor large-scale cultivation of cell wall-deficient mutant strains has many associated economic risks of failure to produce effective quantities of product. A reversion from the mutant strain to its WT, contamination of the mutant strain culture by its WT or invasion by grazers would result in rapid reduction or disappearance of the mutant. Therefore, while industrial outdoor cultivation of cell wall-deficient mutant strains does not present a significant environmental risk to its WT in an escape scenario, the fragility of the mutant strain could impact on overall industrial productivity. The WT strain would most likely outcompete the mutant in competitive cultures sharing the same resources.

In order to further investigate the level of industrial risk, productivity from a total biomass and total TAG accumulation perspective was quantified during co-culture experiments, over a range of nitrogen concentrations. A RSM was fit to data on the maximum biomass (*K*, estimated from each of the three replicate time series) and TAG concentrations were calculated, per cell, during the stationary phase to assess biomass and TAG accumulation over time as a function of NH_4_Cl and initial WT:mutant cell number ratio. In accordance with the monoculture experiments (Fig. 1), the maximum *K* was observed when the mutant strain was grown alone at higher NH_4_Cl concentrations. There was no significant difference between the *K* at 200 and 375 mg L^−1^ NH_4_Cl, which corroborated the monoculture experiments. In agreement with the extensive literature on lipid accumulation in *C. reinhardtii*, our study shows that in stationary phase net growth halts and accumulation of TAGs begins (e.g. Siaut et al. 2011). TAGs accumulated in nitrogen deplete conditions when biomass accumulation stopped after 72 h. To analyse this accumulation, TAGs per million cells were plotted in order to normalise TAG content with biomass production (Fig. 4). Unsurprisingly, TAG accumulation was the highest, per million cells, with the 100% mutant strain at the lowest nitrogen concentration (50 mg L^−1^ NH_4_Cl). However, the industrial risk of reduced productivity was shown in the co-cultures. Due to the success of the WT growth compared to the mutant strain, TAG productivity can be reduced up to 60% (Fig. 4).

 The aim of this study was to compare the growth rates of a *C. reinhardtii* WT and mutant strain in monocultures and in competition (co-cultures) to evaluate the risk of the mutant strain escaping into the environment, as well as the industrial risk of losing the desirable phenotype of the mutant. The response surface design applied suggests relatively little environmental risk if the mutant escapes the cultivation vessel, but a potentially significant industrial productivity loss if a WT equivalent is introduced. Ultimately, the experimental approach is suggested as a predictor of environmental and industrial risk as the experiments were based on laboratory tests only and under specific conditions. A more comprehensive study of environmental risks would require incorporating the complexities associated with ecosystems, including increased biodiversity and changing environmental conditions.

## Additional file

**Additional file 1.** Detergent based lysis to differentiate *C. reinhardtii* WT from mutant strain.

## Abbreviations

μ: exponential growth rate
K: carrying capacity
RSM: response surface model
TAG: triglyceride
WT: wild-type
