# Supplementary material for: Competitive growth experiments with a high-lipid Chlamydomonas reinhardtii mutant strain and its wild-type to predict industrial and ecological risks
Source: AMB Express. 2017 Jan 3;7:10. doi: 10.1186/s13568-016-0305-x (PMC5209313; doi:10.1186/s13568-016-0305-x)
Supplement: Supplementary file 1 — Additional file 1. Detergent based lysis to differentiate C. reinhardtii WT from mutant strain. [file 13568_2016_305_MOESM1_ESM.pdf]

Journal: AMB express

Competitive growth experiments with a high-lipid *Chlamydomonas reinhardtii* mutant strain and its wild-type to predict industrial and ecological risks

David A. Russo[a*], Andrew P. Beckerman[b], Jagroop Pandhal[c]

[a]AlgaeCytes Ltd., Discovery Park House, Discovery Park, Ramsgate Road, Sandwich, CT13 9ND

[b]Department of Animal and Plant Sciences, University of Sheffield, Alfred Denny Building, Western Bank, Sheffield, S10 2TN, UK

[c]Department of Chemical and Biological Engineering, University of Sheffield, Mappin Street, Sheffield S1 3JD, United Kingdom

*corresponding author

Email: davidarusso@gmail.com

Telephone: +44 (0)7453512221

Additional file 1

Detergent based lysis to differentiate C. reinhardtii WT from mutant strain

The WT and mutant *C. reinhardtii* strains are phenotypically very different (e.g. mutant strain lacks flagellum and cell wall) however, under an optical microscope, they are undistinguishable. Therefore, a simple and quick technique had to be devised in order to count cells for each strain separately in a mixed culture. The initial description of the *C. reinhardtii* cell wall deficient strains describes them as flat, liquid colonies on agar, and with cells that lyse in a 1% solution of a non-ionic detergent NP-40. Based on this mutant and WT cells were mixed in known cell number ratios (100:0, 75:25, 50:50, 75:25 and 0:100) and an equal volume of Triton X-100 2% (v/v) was added to the cell suspensions in order to have a working concentration of 1%. The cell suspensions were counted and incubated for ten minutes with re-counts at five and ten minutes. Based on the results at five minutes the observed cell counts were plotted against the expected cell counts to determine precision and accuracy (Fig. A1). The observed cell counts are both precise and accurate ($r^2 = 0.98$).

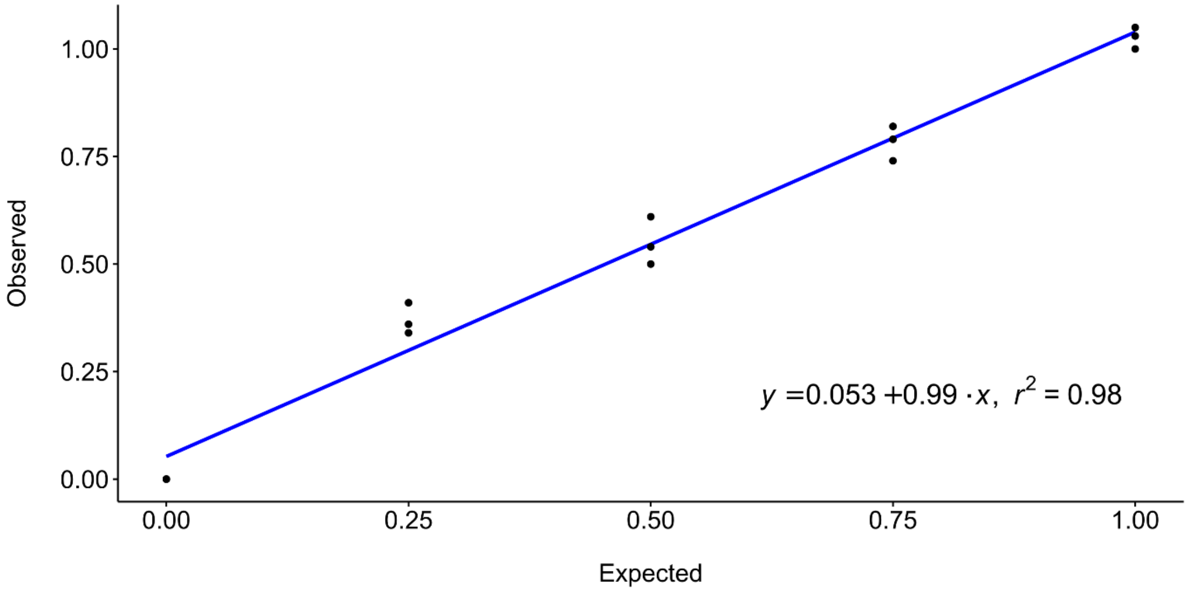

Figure A1. Plot of observed cell counts against expected cell counts after a five minute incubation period. The percentages on both axes represent the percentage of initial WT cells in the mixtures.

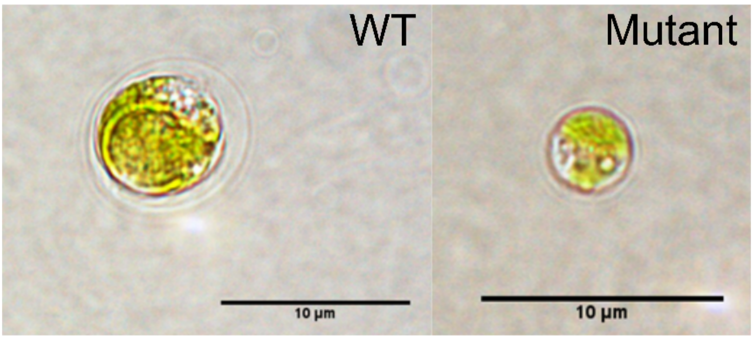

Figure A2. Optical microscopy pictures (1000x magnification) demonstrating the size difference between *C. reinhardtii* WT cells (left) and mutant cells (right).
